# 1,2-Bis(2-furylmethyl­ene)hydrazine

**DOI:** 10.1107/S1600536808030729

**Published:** 2008-09-27

**Authors:** Qi Ma, Li-Ping Lu, Miao-Li Zhu

**Affiliations:** a College of Chemistry and Chemical Engineering, Shanxi Datong University, Datong, Shanxi 037009, People’s Republic of China; b Institute of Molecular Science, Key Laboratory of Chemical Biology and Molecular Engineering of the Education Ministry, Shanxi University, Taiyuan, Shanxi 030006, People’s Republic of China

## Abstract

Crystals of the title compound, C_10_H_8_N_2_O_2_, were obtained from a condensation reaction of hydrazine hydrate with furfural. In the crystal structure, the mol­ecule is centrosymmetric and almost planar and the furan rings are parallel by symmetry.

## Related literature

For background, see: Casellato & Vigato (1977 ▶); For related structures, see: Fan *et al.* (2008 ▶); Shan *et al.* (2004 ▶); Shan, Tian *et al.* (2008 ▶); Shan, Wang *et al.* (2008 ▶).
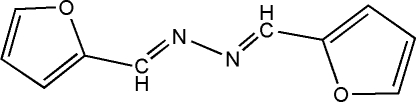


## Experimental

### 

#### Crystal data

C_10_H_8_N_2_O_2_
*M* *_r_* = 188.18
Orthorhombic, 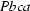

*a* = 6.877 (2) Å
*b* = 8.996 (3) Å
*c* = 15.171 (4) Å
*V* = 938.6 (5) Å^3^
*Z* = 4
Mo *K*α radiation
μ = 0.10 mm^−1^
*T* = 298 (2) K
0.50 × 0.40 × 0.40 mm

#### Data collection

Bruker SMART 1K CCD diffractometer
Absorption correction: multi-scan (*SADABS*; Sheldrick, 2000 ▶) *T*_min_ = 0.954, *T*_max_ = 0.963
4129 measured reflections
829 independent reflections
677 reflections with *I* > 2σ(*I*)
*R*_int_ = 0.081

#### Refinement

*R*[*F* ^2^ > 2σ(*F* ^2^)] = 0.039
*wR*(*F* ^2^) = 0.105
*S* = 1.05
829 reflections
65 parameters
H-atom parameters constrained
Δρ_max_ = 0.16 e Å^−3^
Δρ_min_ = −0.25 e Å^−3^

### 

Data collection: *SMART* (Bruker, 2000 ▶); cell refinement: *SAINT* (Bruker, 2000 ▶); data reduction: *SAINT*; program(s) used to solve structure: *SHELXS97* (Sheldrick, 2008 ▶); program(s) used to refine structure: *SHELXL97* (Sheldrick, 2008 ▶); molecular graphics: *SHELXTL/PC* (Sheldrick, 2008 ▶); software used to prepare material for publication: *SHELXTL/PC*.

## Supplementary Material

Crystal structure: contains datablocks I, global. DOI: 10.1107/S1600536808030729/hb2802sup1.cif

Structure factors: contains datablocks I. DOI: 10.1107/S1600536808030729/hb2802Isup2.hkl

Additional supplementary materials:  crystallographic information; 3D view; checkCIF report

## Figures and Tables

**Fig. 1.**
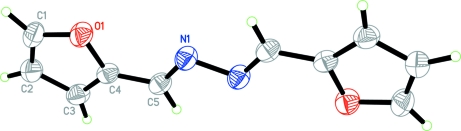
A view of the molecular structure of (I) with displacement ellipsoids for the non-hydrogen atoms drawn at the 30% probability level (arbitrary spheres for the H atoms). The unlabelled atoms are generated by the symmetry operation (1-x, 2-y, -z).

## supplementary crystallographic information

### Comment

Schiff bases have been studied for decades (Casellato & Vigato 1977) and they are still one of the most prevalent mixed-donor ligands in coordination chemistry, with numerous applications including single-molecule magnetism, materials science and catalysis. Here, the synthsis and crystal structure of the title compound (I) are reported.

The molecule of (I) is centrosymmetric, with the midpoint of the N—N bond located on the inversion center. The C5—N1 double bond distance of 1.272 (2) Å is shorter than the C═N bond distance found in related hydrazone structures, *i.e.* 1.295 (2) Å in (*E*)-3-methoxyacetophenone 4-nitrophenylhydrazone (Fan *et al.*, 2008), 1.298 (2) Å in (E)-2-furylmethylketone 2,4-dinitrophenylhydrazone (Shan, Tian *et al.*, 2008) and 1.293 (2) Å in benzylideneacetone 2,4-dinitrophenylhydrazone (Shan *et al.*, 2004).  It is indistinguishable from the length of 1.273 (1) Å in 2-methylbenzaldehyde 2-methylbenzylidenehydrazone (Shan, Wang *et al.*, 2008).

### Experimental

Hydrazine hydrate (35% solution in water, 0.71 g, 5 mmol) and furfural (0.96 g, 10 mmol) were mixed, at the same time adding 2 or 3 drops of formic acid, and stirred at room temperature in 30 ml of ethanol solution for 2 days, and then the filtrate was kept open to slowly evaporate for a few days, depositing yellow blocks of (I).

### Refinement

The H atoms attached were placed in geometrically idealized positions (C—H = 0.93 Å) and refined as riding with U_iso_(H) = 1.2U_eq_(C).

### Crystal data

**Table:**

C_10_H_8_N_2_O_2_	*F*(000) = 392
*M**_r_* = 188.18	*D*_x_ = 1.332 Mg m^−^^3^
Orthorhombic, *P**b**c**a*	Mo *K*α radiation, λ = 0.71073 Å
Hall symbol: -P 2ac 2ab	Cell parameters from 5207 reflections
*a* = 6.877 (2) Å	θ = 2.6–25.3°
*b* = 8.996 (3) Å	µ = 0.10 mm^−^^1^
*c* = 15.171 (4) Å	*T* = 298 K
*V* = 938.6 (5) Å^3^	Block, yellow
*Z* = 4	0.50 × 0.40 × 0.40 mm

### Data collection

**Table:**

Bruker SMART 1K CCD diffractometer	829 independent reflections
Radiation source: fine-focus sealed tube	677 reflections with *I* > 2σ(*I*)
graphite	*R*_int_ = 0.081
ω scans	θ_max_ = 25.3°, θ_min_ = 2.6°
Absorption correction: multi-scan (SADABS; Sheldrick, 2000)	*h* = −8→8
*T*_min_ = 0.954, *T*_max_ = 0.963	*k* = −10→10
4129 measured reflections	*l* = −18→9

### Refinement

**Table:**

Refinement on *F*^2^	Primary atom site location: structure-invariant direct methods
Least-squares matrix: full	Secondary atom site location: difference Fourier map
*R*[*F*^2^ > 2σ(*F*^2^)] = 0.039	Hydrogen site location: inferred from neighbouring sites
*wR*(*F*^2^) = 0.105	H-atom parameters constrained
*S* = 1.05	*w* = 1/[σ^2^(*F*_o_^2^) + (0.0662*P*)^2^] where *P* = (*F*_o_^2^ + 2*F*_c_^2^)/3
829 reflections	(Δ/σ)_max_ < 0.001
65 parameters	Δρ_max_ = 0.16 e Å^−^^3^
0 restraints	Δρ_min_ = −0.25 e Å^−^^3^

### Special details

**Table:**

Geometry. All e.s.d.'s (except the e.s.d. in the dihedral angle between two l.s. planes) are estimated using the full covariance matrix. The cell e.s.d.'s are taken into account individually in the estimation of e.s.d.'s in distances, angles and torsion angles; correlations between e.s.d.'s in cell parameters are only used when they are defined by crystal symmetry. An approximate (isotropic) treatment of cell e.s.d.'s is used for estimating e.s.d.'s involving l.s. planes.
Refinement. Refinement of *F*^2^ against ALL reflections. The weighted *R*-factor *wR* and goodness of fit *S* are based on *F*^2^, conventional *R*-factors *R* are based on *F*, with *F* set to zero for negative *F*^2^. The threshold expression of *F*^2^ > σ(*F*^2^) is used only for calculating *R*-factors(gt) *etc*. and is not relevant to the choice of reflections for refinement. *R*-factors based on *F*^2^ are statistically about twice as large as those based on *F*, and *R*- factors based on ALL data will be even larger.

### Fractional atomic coordinates and isotropic or equivalent isotropic displacement parameters (Å^2^)

**Table:**

	*x*	*y*	*z*	*U*_iso_*/*U*_eq_	
C1	−0.0467 (2)	0.88870 (18)	0.18436 (11)	0.0736 (5)	
H1	−0.0937	0.8074	0.2157	0.088*	
C2	−0.1336 (2)	1.01970 (19)	0.18094 (10)	0.0719 (5)	
H2	−0.2492	1.0466	0.2084	0.086*	
C3	−0.0168 (2)	1.10963 (17)	0.12769 (10)	0.0642 (5)	
H3	−0.0400	1.2085	0.1131	0.077*	
C4	0.13456 (19)	1.02755 (14)	0.10149 (8)	0.0529 (4)	
C5	0.2964 (2)	1.06191 (15)	0.04628 (9)	0.0566 (4)	
H5	0.3049	1.1570	0.0225	0.068*	
N1	0.42950 (18)	0.96866 (13)	0.02805 (8)	0.0613 (4)	
O1	0.12017 (16)	0.88799 (10)	0.13632 (7)	0.0669 (4)	

### Atomic displacement parameters (Å^2^)

**Table:**

	*U*^11^	*U*^22^	*U*^33^	*U*^12^	*U*^13^	*U*^23^
C1	0.0695 (10)	0.0698 (11)	0.0815 (11)	−0.0135 (8)	0.0114 (9)	0.0137 (8)
C2	0.0637 (10)	0.0800 (12)	0.0721 (11)	0.0049 (8)	0.0084 (7)	0.0085 (8)
C3	0.0722 (11)	0.0589 (10)	0.0616 (9)	0.0093 (7)	0.0038 (7)	0.0082 (7)
C4	0.0639 (9)	0.0452 (8)	0.0496 (7)	−0.0049 (6)	−0.0044 (6)	0.0007 (6)
C5	0.0690 (10)	0.0485 (8)	0.0522 (8)	−0.0055 (7)	0.0007 (7)	0.0013 (6)
N1	0.0649 (8)	0.0541 (8)	0.0649 (8)	−0.0033 (6)	0.0084 (5)	0.0045 (5)
O1	0.0698 (8)	0.0479 (7)	0.0830 (8)	−0.0029 (5)	0.0078 (5)	0.0057 (4)

### Geometric parameters (Å, °)

**Table:**

C1—C2	1.322 (2)	C3—H3	0.9300
C1—O1	1.359 (2)	C4—O1	1.3657 (16)
C1—H1	0.9300	C4—C5	1.427 (2)
C2—C3	1.397 (2)	C5—N1	1.2721 (18)
C2—H2	0.9300	C5—H5	0.9300
C3—C4	1.3364 (19)	N1—N1^i^	1.408 (2)
			
C2—C1—O1	111.39 (14)	C3—C4—O1	109.66 (12)
C2—C1—H1	124.3	C3—C4—C5	131.47 (13)
O1—C1—H1	124.3	O1—C4—C5	118.87 (12)
C1—C2—C3	106.18 (15)	N1—C5—C4	123.10 (13)
C1—C2—H2	126.9	N1—C5—H5	118.4
C3—C2—H2	126.9	C4—C5—H5	118.4
C4—C3—C2	107.45 (14)	C5—N1—N1^i^	111.29 (15)
C4—C3—H3	126.3	C1—O1—C4	105.32 (12)
C2—C3—H3	126.3		
			
O1—C1—C2—C3	−0.05 (19)	O1—C4—C5—N1	−0.4 (2)
C1—C2—C3—C4	0.23 (18)	C4—C5—N1—N1^i^	179.64 (14)
C2—C3—C4—O1	−0.32 (17)	C2—C1—O1—C4	−0.14 (18)
C2—C3—C4—C5	179.42 (15)	C3—C4—O1—C1	0.29 (16)
C3—C4—C5—N1	179.91 (15)	C5—C4—O1—C1	−179.49 (12)

Symmetry codes: (i) −*x*+1, −*y*+2, −*z*.
